# 3-Acetyl-4-hy­droxy-6,7-dimethyl-2*H*-chromen-2-one

**DOI:** 10.1107/S1600536810045010

**Published:** 2010-11-13

**Authors:** Mohammad Asad, Chuan-Wei Oo, Hasnah Osman, Jia Hao Goh, Hoong-Kun Fun

**Affiliations:** a School of Chemical Sciences, Universiti Sains Malaysia, 11800 USM, Penang, Malaysia; b X-ray Crystallography Unit, School of Physics, Universiti Sains Malaysia, 11800 USM, Penang, Malaysia

## Abstract

In the title coumarin derivative, C_13_H_12_O_4_, the 2*H*-chromene ring system is essentially planar [maximum deviation = 0.047 (1) Å]. An intra­molecular hydrogen bond is observed between the hy­droxy and the ketonic O atoms. In the crystal, pairs of inter­molecular C—H⋯O hydrogen bonds link inversion-related mol­ecules into dimers. Additional inter­molecular C—H⋯O hydrogen bonds further inter­connect these dimers into two-dimensional arrays incorporating *R*_2_^2^(9) ring motifs.

## Related literature

For general background to and applications of coumarin derivatives, see: Eisenhauer & Link (1953 ▶); Franz *et al.* (1981 ▶); Frontiera *et al.* (2009 ▶); Maurer & Arlt (1998 ▶). Tamura *et al.* (1982 ▶); Wang *et al.* (2007 ▶). For graph-set theory of hydrogen-bond ring motifs, see: Bernstein *et al.* (1995 ▶). For bond-length data, see: Allen *et al.* (1987 ▶). For a related coumaric structure, see: Mechi *et al.* (2009 ▶). For the stability of the temperature controller used in the data collection, see: Cosier & Glazer (1986 ▶).
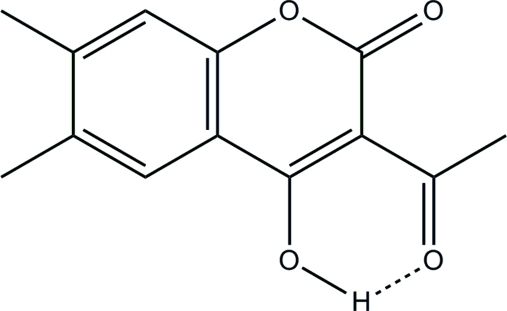


## Experimental

### 

#### Crystal data

C_13_H_12_O_4_
*M* *_r_* = 232.23
Monoclinic, 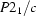

*a* = 3.9491 (4) Å
*b* = 12.1359 (11) Å
*c* = 22.101 (2) Å
β = 90.563 (1)°
*V* = 1059.16 (17) Å^3^
*Z* = 4
Mo *K*α radiation
μ = 0.11 mm^−1^
*T* = 100 K
0.32 × 0.19 × 0.13 mm

#### Data collection

Bruker APEXII DUO CCD area-detector diffractometer
Absorption correction: multi-scan (*SADABS*; Bruker, 2009 ▶) *T*_min_ = 0.966, *T*_max_ = 0.986
13172 measured reflections
3139 independent reflections
2539 reflections with *I* > 2σ(*I*)
*R*_int_ = 0.030

#### Refinement

*R*[*F* ^2^ > 2σ(*F* ^2^)] = 0.048
*wR*(*F* ^2^) = 0.148
*S* = 1.05
3139 reflections
161 parameters
H atoms treated by a mixture of independent and constrained refinement
Δρ_max_ = 0.64 e Å^−3^
Δρ_min_ = −0.28 e Å^−3^

### 

Data collection: *APEX2* (Bruker, 2009 ▶); cell refinement: *SAINT* (Bruker, 2009 ▶); data reduction: *SAINT*; program(s) used to solve structure: *SHELXTL* (Sheldrick, 2008 ▶); program(s) used to refine structure: *SHELXTL*; molecular graphics: *SHELXTL*; software used to prepare material for publication: *SHELXTL* and *PLATON* (Spek, 2009 ▶).

## Supplementary Material

Crystal structure: contains datablocks global, I. DOI: 10.1107/S1600536810045010/rz2512sup1.cif

Structure factors: contains datablocks I. DOI: 10.1107/S1600536810045010/rz2512Isup2.hkl

Additional supplementary materials:  crystallographic information; 3D view; checkCIF report

## Figures and Tables

**Table 1:** Hydrogen-bond geometry (Å, °)

*D*—H⋯*A*	*D*—H	H⋯*A*	*D*⋯*A*	*D*—H⋯*A*
O2—H1*O*2⋯O3	1.277 (18)	1.183 (18)	2.4299 (14)	162.0 (16)
C6—H6*A*⋯O3^i^	0.93	2.58	3.4603 (17)	159
C11—H11*B*⋯O2^ii^	0.96	2.59	3.5458 (18)	172
C12—H12*A*⋯O4^iii^	0.96	2.53	3.4751 (17)	168

Symmetry codes: (i) 
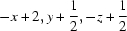
; (ii) 
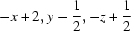
; (iii) 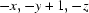.

**Fig. 1.**
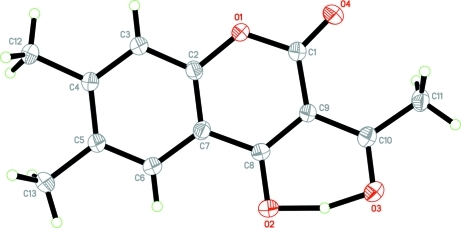
The molecular structure of the title coumarin compound, showing 50 % probability displacement ellipsoids for non-H atoms and the atom-numbering scheme.

**Fig. 2.**
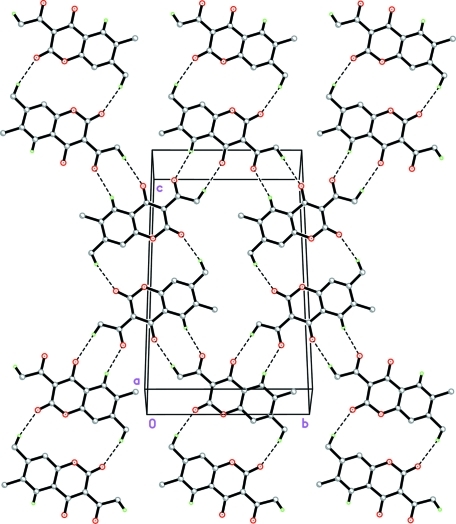
The crystal structure of the title coumarin compound, viewed along an arbitrary axis, showing dimers being linked into a two-dimensional array. H atoms not involved in intermolecular hydrogen bonds (dashed lines) have been omitted for clarity.

## supplementary crystallographic information

### Comment

We report here a new 4-hydroxycoumarin derivative, which has been synthesized by acetylation process (Eisenhauer & Link, 1953). Recently, coumarin and its derivatives have been extensively used in industrial products as dyes/laser materials (Wang *et al.*, 2007), photosensitizers (Frontiera *et al.*, 2009), pesticides (Franz *et al.*, 1981), in pharmacology (Maurer  & Arlt, 1998) and in enzymology as biological probes (Tamura *et al.*, 1982).

In the title coumarin compound, (Fig. 1), the 2*H*-chromene ring system (C1-C9/O1) is essentially planar, as indicated by the maximum deviation of -0.047 (1) Å at atom C1. Bond length of C10═O3 [1.2590 (16) Å] is longer than normal value due to the delocalization of atom H1O2 between the hydroxyl oxygen atom (O2) and the ketonic oxygen atom (O3), as observed in a related structure (Mechi *et al.*, 2009). However, the bond lengths of O2—H1O2 = 1.277 (18) and O3—H1O2 = 1.183 (18) Å are inconsistent with the respective values observed in Mechi *et al.*, 2009. All other bond lengths (Allen *et al.*, 1987) and angles are within normal ranges. In the crystal structure, (Fig. 2), pairs of intermolecular C12—H12A···O4 hydrogen bonds (Table 1) link inversion-related molecules into dimers, producing *R*_2_^2^(16) ring motifs (Bernstein *et al.*, 1995). Intermolecular C6—H16A···O3 and C11—H11B···O2 hydrogen bonds (Table 1) further interconnect these dimers into two-dimensional arrays incorporating *R*_2_^2^(9) hydrogen bond ring motifs (Bernstein *et al.*, 1995).

### Experimental

Acetyl chloride (1 ml) was added to a solution of 4-hydroxy-6,7-dimethylcoumarin (5.2 mmol, 1.0 g) in pyridine (10 ml) which contains piperidine (one drop) on ice bath. The reaction mixture was kept at room temperature for 7 days. The solution was then poured into ice-cold water and hydrochloric acid was added to afford the precipitate, which was washed with water, dried and recrystallized from ethanol to get the pure title compound in 70% yield.

### Refinement

Atom  H1O2 was located in a difference Fourier map and allowed to refine freely. The remaining H atoms were placed in their calculated positions, with C—H = 0.93–0.96 Å, and refined using a riding model, with *U*_iso_ = 1.2 or 1.5 *U*_eq_(C). The rotating group model was applied to the methyl groups.

### Crystal data

**Table:**

C_13_H_12_O_4_	*F*(000) = 488
*M**_r_* = 232.23	*D*_x_ = 1.456 Mg m^−^^3^
Monoclinic, *P*2_1_/*c*	Mo *K*α radiation, λ = 0.71073 Å
Hall symbol: -P 2ybc	Cell parameters from 5636 reflections
*a* = 3.9491 (4) Å	θ = 3.2–30.2°
*b* = 12.1359 (11) Å	µ = 0.11 mm^−^^1^
*c* = 22.101 (2) Å	*T* = 100 K
β = 90.563 (1)°	Block, brown
*V* = 1059.16 (17) Å^3^	0.32 × 0.19 × 0.13 mm
*Z* = 4	

### Data collection

**Table:**

Bruker APEXII DUO CCD area-detector diffractometer	3139 independent reflections
Radiation source: fine-focus sealed tube	2539 reflections with *I* > 2σ(*I*)
graphite	*R*_int_ = 0.030
φ and ω scans	θ_max_ = 30.2°, θ_min_ = 3.2°
Absorption correction: multi-scan (*SADABS*; Bruker, 2009)	*h* = −5→5
*T*_min_ = 0.966, *T*_max_ = 0.986	*k* = −17→17
13172 measured reflections	*l* = −29→31

### Refinement

**Table:**

Refinement on *F*^2^	Primary atom site location: structure-invariant direct methods
Least-squares matrix: full	Secondary atom site location: difference Fourier map
*R*[*F*^2^ > 2σ(*F*^2^)] = 0.048	Hydrogen site location: inferred from neighbouring sites
*wR*(*F*^2^) = 0.148	H atoms treated by a mixture of independent and constrained refinement
*S* = 1.05	*w* = 1/[σ^2^(*F*_o_^2^) + (0.0758*P*)^2^ + 0.5033*P*] where *P* = (*F*_o_^2^ + 2*F*_c_^2^)/3
3139 reflections	(Δ/σ)_max_ < 0.001
161 parameters	Δρ_max_ = 0.64 e Å^−^^3^
0 restraints	Δρ_min_ = −0.28 e Å^−^^3^

### Special details

**Table:**

Experimental. The crystal was placed in the cold stream of an Oxford Cryosystems Cobra open-flow nitrogen cryostat (Cosier & Glazer, 1986) operating at 100.0 (1)K.
Geometry. All esds (except the esd in the dihedral angle between two l.s. planes) are estimated using the full covariance matrix. The cell esds are taken into account individually in the estimation of esds in distances, angles and torsion angles; correlations between esds in cell parameters are only used when they are defined by crystal symmetry. An approximate (isotropic) treatment of cell esds is used for estimating esds involving l.s. planes.
Refinement. Refinement of F^2^ against ALL reflections. The weighted R-factor wR and goodness of fit S are based on F^2^, conventional R-factors R are based on F, with F set to zero for negative F^2^. The threshold expression of F^2^ > 2sigma(F^2^) is used only for calculating R-factors(gt) etc. and is not relevant to the choice of reflections for refinement. R-factors based on F^2^ are statistically about twice as large as those based on F, and R- factors based on ALL data will be even larger.

### Fractional atomic coordinates and isotropic or equivalent isotropic displacement parameters (Å^2^)

**Table:**

	*x*	*y*	*z*	*U*_iso_*/*U*_eq_	
O1	0.2856 (2)	0.47223 (7)	0.06747 (4)	0.0226 (2)	
O2	0.8349 (3)	0.53534 (8)	0.22188 (4)	0.0257 (2)	
O3	0.8887 (3)	0.33852 (8)	0.23973 (5)	0.0279 (2)	
O4	0.2728 (3)	0.29458 (8)	0.08591 (5)	0.0282 (2)	
C1	0.3765 (3)	0.38338 (10)	0.10303 (6)	0.0207 (2)	
C2	0.3684 (3)	0.57902 (10)	0.08348 (6)	0.0194 (2)	
C3	0.2653 (3)	0.66119 (10)	0.04395 (6)	0.0203 (2)	
H3A	0.1509	0.6432	0.0083	0.024*	
C4	0.3335 (3)	0.77060 (10)	0.05780 (6)	0.0193 (2)	
C5	0.5009 (3)	0.79852 (10)	0.11250 (6)	0.0198 (2)	
C6	0.6079 (3)	0.71492 (10)	0.15068 (6)	0.0203 (2)	
H6A	0.7232	0.7324	0.1863	0.024*	
C7	0.5447 (3)	0.60395 (10)	0.13639 (6)	0.0192 (2)	
C8	0.6577 (3)	0.51334 (10)	0.17322 (5)	0.0193 (2)	
C9	0.5807 (3)	0.40436 (10)	0.15620 (5)	0.0184 (2)	
C10	0.7117 (3)	0.31530 (11)	0.19379 (6)	0.0218 (3)	
C11	0.6506 (4)	0.19714 (11)	0.18029 (7)	0.0259 (3)	
H11B	0.7690	0.1525	0.2094	0.039*	
H11C	0.7309	0.1805	0.1405	0.039*	
H11D	0.4124	0.1820	0.1822	0.039*	
C12	0.2309 (3)	0.85786 (11)	0.01304 (6)	0.0249 (3)	
H12A	0.0781	0.8268	−0.0164	0.037*	
H12B	0.4284	0.8854	−0.0069	0.037*	
H12C	0.1207	0.9171	0.0338	0.037*	
C13	0.5604 (4)	0.91704 (11)	0.12989 (7)	0.0264 (3)	
H13A	0.6985	0.9200	0.1658	0.040*	
H13B	0.3471	0.9522	0.1373	0.040*	
H13C	0.6735	0.9544	0.0976	0.040*	
H1O2	0.886 (4)	0.4360 (15)	0.2387 (8)	0.029 (4)*	

### Atomic displacement parameters (Å^2^)

**Table:**

	*U*^11^	*U*^22^	*U*^33^	*U*^12^	*U*^13^	*U*^23^
O1	0.0297 (5)	0.0181 (4)	0.0201 (4)	−0.0013 (3)	−0.0067 (3)	0.0018 (3)
O2	0.0335 (5)	0.0222 (5)	0.0210 (5)	−0.0004 (4)	−0.0084 (4)	−0.0013 (4)
O3	0.0345 (5)	0.0247 (5)	0.0244 (5)	0.0003 (4)	−0.0086 (4)	0.0041 (4)
O4	0.0363 (5)	0.0201 (5)	0.0282 (5)	−0.0049 (4)	−0.0071 (4)	−0.0018 (4)
C1	0.0241 (5)	0.0180 (5)	0.0201 (6)	−0.0002 (4)	−0.0010 (4)	0.0013 (4)
C2	0.0222 (5)	0.0157 (5)	0.0203 (6)	0.0000 (4)	0.0006 (4)	0.0000 (4)
C3	0.0235 (5)	0.0182 (5)	0.0191 (6)	−0.0003 (4)	−0.0017 (4)	0.0001 (4)
C4	0.0213 (5)	0.0169 (5)	0.0198 (6)	0.0010 (4)	−0.0008 (4)	0.0015 (4)
C5	0.0222 (5)	0.0173 (5)	0.0197 (6)	0.0000 (4)	−0.0006 (4)	0.0000 (4)
C6	0.0225 (5)	0.0202 (5)	0.0182 (6)	−0.0002 (4)	−0.0020 (4)	0.0002 (4)
C7	0.0214 (5)	0.0182 (5)	0.0179 (6)	0.0014 (4)	0.0000 (4)	0.0019 (4)
C8	0.0214 (5)	0.0201 (5)	0.0163 (5)	−0.0002 (4)	−0.0016 (4)	−0.0002 (4)
C9	0.0211 (5)	0.0163 (5)	0.0177 (5)	0.0004 (4)	−0.0007 (4)	0.0013 (4)
C10	0.0229 (5)	0.0214 (6)	0.0212 (6)	0.0006 (4)	0.0000 (4)	0.0029 (4)
C11	0.0293 (6)	0.0178 (6)	0.0304 (7)	0.0002 (4)	−0.0035 (5)	0.0051 (5)
C12	0.0304 (6)	0.0196 (6)	0.0245 (6)	0.0015 (5)	−0.0060 (5)	0.0039 (5)
C13	0.0331 (6)	0.0182 (6)	0.0279 (7)	−0.0021 (5)	−0.0050 (5)	−0.0015 (5)

### Geometric parameters (Å, °)

**Table:**

O1—C1	1.3797 (15)	C6—C7	1.4050 (17)
O1—C2	1.3819 (15)	C6—H6A	0.9300
O2—C8	1.3048 (15)	C7—C8	1.4366 (17)
O2—H1O2	1.277 (18)	C8—C9	1.4074 (17)
O3—C10	1.2590 (16)	C9—C10	1.4550 (17)
O3—H1O2	1.183 (18)	C10—C11	1.4840 (18)
O4—C1	1.2121 (15)	C11—H11B	0.9600
C1—C9	1.4415 (17)	C11—H11C	0.9600
C2—C3	1.3843 (17)	C11—H11D	0.9600
C2—C7	1.3884 (17)	C12—H12A	0.9600
C3—C4	1.3884 (17)	C12—H12B	0.9600
C3—H3A	0.9300	C12—H12C	0.9600
C4—C5	1.4133 (17)	C13—H13A	0.9600
C4—C12	1.5022 (17)	C13—H13B	0.9600
C5—C6	1.3831 (17)	C13—H13C	0.9600
C5—C13	1.5066 (17)		
			
C1—O1—C2	121.83 (10)	C9—C8—C7	120.19 (11)
C8—O2—H1O2	97.4 (8)	C8—C9—C1	120.10 (11)
C10—O3—H1O2	101.7 (9)	C8—C9—C10	118.09 (11)
O4—C1—O1	115.58 (11)	C1—C9—C10	121.82 (11)
O4—C1—C9	126.59 (12)	O3—C10—C9	119.06 (12)
O1—C1—C9	117.83 (10)	O3—C10—C11	117.79 (11)
O1—C2—C3	116.52 (11)	C9—C10—C11	123.15 (11)
O1—C2—C7	122.39 (11)	C10—C11—H11B	109.5
C3—C2—C7	121.09 (11)	C10—C11—H11C	109.5
C2—C3—C4	119.64 (11)	H11B—C11—H11C	109.5
C2—C3—H3A	120.2	C10—C11—H11D	109.5
C4—C3—H3A	120.2	H11B—C11—H11D	109.5
C3—C4—C5	120.40 (11)	H11C—C11—H11D	109.5
C3—C4—C12	118.59 (11)	C4—C12—H12A	109.5
C5—C4—C12	121.00 (11)	C4—C12—H12B	109.5
C6—C5—C4	118.91 (11)	H12A—C12—H12B	109.5
C6—C5—C13	119.92 (11)	C4—C12—H12C	109.5
C4—C5—C13	121.17 (11)	H12A—C12—H12C	109.5
C5—C6—C7	120.90 (11)	H12B—C12—H12C	109.5
C5—C6—H6A	119.6	C5—C13—H13A	109.5
C7—C6—H6A	119.6	C5—C13—H13B	109.5
C2—C7—C6	118.99 (11)	H13A—C13—H13B	109.5
C2—C7—C8	117.43 (11)	C5—C13—H13C	109.5
C6—C7—C8	123.57 (11)	H13A—C13—H13C	109.5
O2—C8—C9	121.68 (11)	H13B—C13—H13C	109.5
O2—C8—C7	118.12 (11)		
			
C2—O1—C1—O4	176.00 (11)	C5—C6—C7—C2	−0.86 (19)
C2—O1—C1—C9	−3.75 (17)	C5—C6—C7—C8	178.13 (11)
C1—O1—C2—C3	179.59 (11)	C2—C7—C8—O2	177.43 (11)
C1—O1—C2—C7	−0.62 (18)	C6—C7—C8—O2	−1.57 (19)
O1—C2—C3—C4	178.64 (11)	C2—C7—C8—C9	−1.77 (18)
C7—C2—C3—C4	−1.15 (19)	C6—C7—C8—C9	179.23 (11)
C2—C3—C4—C5	−1.35 (18)	O2—C8—C9—C1	178.30 (11)
C2—C3—C4—C12	177.66 (11)	C7—C8—C9—C1	−2.53 (18)
C3—C4—C5—C6	2.68 (18)	O2—C8—C9—C10	−1.45 (18)
C12—C4—C5—C6	−176.30 (11)	C7—C8—C9—C10	177.72 (11)
C3—C4—C5—C13	−176.65 (12)	O4—C1—C9—C8	−174.46 (13)
C12—C4—C5—C13	4.36 (19)	O1—C1—C9—C8	5.26 (17)
C4—C5—C6—C7	−1.57 (19)	O4—C1—C9—C10	5.3 (2)
C13—C5—C6—C7	177.78 (11)	O1—C1—C9—C10	−175.00 (11)
O1—C2—C7—C6	−177.53 (11)	C8—C9—C10—O3	−0.23 (18)
C3—C2—C7—C6	2.25 (19)	C1—C9—C10—O3	−179.98 (12)
O1—C2—C7—C8	3.42 (18)	C8—C9—C10—C11	−179.43 (12)
C3—C2—C7—C8	−176.80 (11)	C1—C9—C10—C11	0.82 (19)

### Hydrogen-bond geometry (Å, °)

**Table:**

*D*—H···*A*	*D*—H	H···*A*	*D*···*A*	*D*—H···*A*
O2—H1O2···O3	1.277 (18)	1.183 (18)	2.4299 (14)	162.0 (16)
C6—H6A···O3^i^	0.93	2.58	3.4603 (17)	159
C11—H11B···O2^ii^	0.96	2.59	3.5458 (18)	172
C12—H12A···O4^iii^	0.96	2.53	3.4751 (17)	168

Symmetry codes: (i) −*x*+2, *y*+1/2, −*z*+1/2; (ii) −*x*+2, *y*−1/2, −*z*+1/2; (iii) −*x*, −*y*+1, −*z*.
